# Evaluation of Ac-Lys^0^(IRDye800CW)Tyr^3^-octreotate as a novel tracer for SSTR_2_-targeted molecular fluorescence guided surgery in meningioma

**DOI:** 10.1007/s11060-021-03739-1

**Published:** 2021-03-26

**Authors:** Bianca M. Dijkstra, Marion de Jong, Marcus C. M. Stroet, Fritz Andreae, Sebastiaan E. Dulfer, Marieke Everts, Schelto Kruijff, Julie Nonnekens, Wilfred F. A. den Dunnen, Frank A. E. Kruyt, Rob J. M. Groen

**Affiliations:** 1 Department of Neurosurgery, University of Groningen, University Medical Center Groningen, Hanzeplein 1, P.O. Box 30.001, 9700 VB Groningen, The Netherlands; 2 Department of Radiology and Nuclear Medicine, Erasmus MC, Rotterdam, The Netherlands; 3 Department of Molecular Genetics, Oncode Institute, Erasmus MC, Rotterdam, The Netherlands; 4 piCHEM Forschungs und EntwicklungsGmbH, Raaba-Grambach, Graz, Austria; 5 Department of Medical Oncology, University of Groningen, University Medical Center Groningen, Groningen, The Netherlands; 6 Department of Surgery, University of Groningen, University Medical Center Groningen, Groningen, The Netherlands; 7 Department of Pathology and Medical Biology, University of Groningen, University Medical Center Groningen, Groningen, The Netherlands

**Keywords:** 800CW-TATE, Intra-operative guidance, Meningioma, Molecular fluorescence guided surgery, Somatostatin receptor subtype 2

## Abstract

**Purpose:**

Meningioma recurrence rates can be reduced by optimizing surgical resection with the use of intraoperative molecular fluorescence guided surgery (MFGS). We evaluated the potential of the fluorescent tracer 800CW-TATE for MFGS using in vitro and in vivo models. It targets somatostatin receptor subtype 2 (SSTR_2_), which is overexpressed in all meningiomas.

**Methods:**

Binding affinity of 800CW-TATE was evaluated using [^177^Lu] Lu-DOTA-Tyr^3^-octreotate displacement assays. Tumor uptake was determined by injecting 800CW-TATE in (SSTR_2_-positive) NCI-H69 or (SSTR_2_-negative) CH-157MN xenograft bearing mice and FMT2500 imaging. SSTR_2_-specific binding was measured by comparing tumor uptake in NCI-H69 and CH-157MN xenografts, blocking experiments and non-targeted IRDye800CW-carboxylate binding. Tracer distribution was analyzed ex vivo, and the tumor-to-background ratio (TBR) was calculated. SSTR_2_ expression was determined by immunohistochemistry (IHC). Lastly, 800CW-TATE was incubated on frozen and fresh meningioma specimens and analyzed by microscopy.

**Results:**

800CW-TATE binding affinity assays showed an IC_50_ value of 72 nM. NCI-H69 xenografted mice showed a TBR of 21.1. 800CW-TATE detection was reduced after co-administration of non-fluorescent DOTA-Tyr^3^-octreotate or administration of IRDye800CW. CH-157MN had no tumor specific tracer staining due to absence of SSTR_2_ expression, thereby serving as a negative control. The tracer bound specifically to SSTR_2_-positive meningioma tissues representing all WHO grades.

**Conclusion:**

800CW-TATE demonstrated sufficient binding affinity, specific SSTR_2_-mediated tumor uptake, a favorable biodistribution, and high TBR. These features make this tracer very promising for use in MFGS and could potentially aid in safer and a more complete meningioma resection, especially in high-grade meningiomas or those at complex anatomical localizations.

**Supplementary Information:**

The online version contains supplementary material available at 10.1007/s11060-021-03739-1.

## Introduction

Meningiomas comprise one third of all intracranial tumors in adults. Although these tumors are most often benign, the compression of intracranial neural structures by a growing meningioma can result in serious neurological signs and symptoms, urging for surgical treatment. The most recent WHO criteria distinguish three grades of meningiomas based on histopathological characteristics: WHO grade I, II and III [1]. Surgical treatment is only curative by complete tumor resection. Unfortunately, anatomical localization and local invasion can pose surgical challenges, leading to high recurrence rates [2–5]. Even in benign (WHO grade I) meningiomas, 10-year progression free survival decreases significantly when comparing incomplete resection to gross total resection: 37% vs 61%, respectively [2]. Molecular fluorescence guided surgery (MFGS) by means of intraoperative guidance could facilitate optimal resection while at the same time aiming to preserve neurological function.

MFGS applies a tumor-targeting tracer that is usually administered intravenously prior to surgery. The tracer consists of two parts: a component specifically binding to a selected tumor-specific biomarker, and a fluorescent dye. The biomarker should be overexpressed on tumor tissue to obtain a sufficiently high tumor-to-background ratio (TBR), which is essential for optimal visualization and thus optimal resection. The fluorescent signal emitted by the tracer allows for intraoperative detection with a neurosurgical microscopic system. IRDye800CW is often applied in tracer development. This dye fluoresces in the near-infrared spectrum, which results in less interference of tissue auto-fluorescence and shows a superior tissue penetration depth. Consequently, such a molecular targeted meningioma-specific tracer may have higher sensitivity, specificity and superior penetration depth compared with currently available dyes such as 5-ALA, ICG or fluorescein.

In the present study, we developed a possible tracer for meningiomas that is directed against the somatostatin receptor subtype 2 (SSTR_2_). We and others previously demonstrated overexpression of SSTR_2_ on all meningioma grades, making it a promising biomarker for binding [6–11]. Tyr^3^-octreotate, a previously generated peptide-analogue with a high affinity for SSTR_2_ [12], served as the meningioma specific binding part. Subsequent labelling with IRDye800CW yielded Ac-Lys^0^(IRDye800CW)Tyr^3^-octreotate (800CW-TATE). Here, we investigated the use of 800CW-TATE for suitability as a tracer in MFGS for meningiomas. Since both octreotate and IRDye800CW-tracers have been already applied in patients [13–16], this tracer is a promising candidate for clinical translation in meningioma surgery.

## Materials and methods

A full description of the materials and methods is available as online resource.

### Cell culture

Small cell lung cancer NCI-H69 (ECACC) and meningioma CH-157MN cells (dr. Gillespie, University of Alabama) were maintained as described previously [17–20].

### Immunohistochemistry on formalin fixed paraffin embedded (FFPE) sections

SSTR_2_ expression in H69 and CH-157MN cells in vitro was determined by analyzing sections of Cellient blocks stained for SSTR_2_ (MAB4224, R&D systems).

Separate consecutive FFPE sections of tissue obtained from animal studies were used for staining with either 4′,6-diamidino-2-phenylindole (DAPI), hematoxylin and eosin (H&E), or SSTR_2_ expression analysis (AC-0162RUO, SanBio) [7].

### Xenograft mice models

H69 (5 million cells) or CH-157MN cells (1.5 million cells) were inoculated in BALB/c-nu mice (Janvier Laboratories) by subcutaneously injecting between the scapulae [17–19].

### Displacement assays

Snap frozen slices of resected H69 xenografts known to abundantly express SSTR_2_ [17–19] were incubated with 10^–9^ M [^177^Lu]Lu-DOTA-Tyr^3^-octreotate ([^177^Lu]Lu-DOTA-TATE; IDB Holland) together with a concentration range of 10^–6^ to 10^–12^ M 800CW-TATE (piCHEM; Online Resource 1), or DOTA-TATE (Bachem). Sections were subsequently washed, and activity was detected with the Cyclone (PerkinElmer). Data was analyzed as described previously [21].

### Tracer uptake, biodistribution and imaging

Mice bearing xenografts (500 mm^3^) were given an intravenous bolus with indicated doses of compounds by retro-orbital injection into the venous sinus under general anesthesia. Tumor uptake of 800CW-TATE was determined by longitudinal imaging for 1, 2, and 4 h post tracer injection using the FMT2500 (PerkinElmer). H69 xenografted mice were injected with either 800CW-TATE (3 µg, 1.36 nmol), DOTA-TATE (3 mg, 2.2 µmol) and 800CW-TATE (3 µg, 1.36 nmol), or IRDye800CW carboxylate (1.5 µg, 1.36 nmol; LI-COR Biosciences). CH-157MN xenograft bearing mice were injected with 800CW-TATE (3 µg, 1.36 nmol). Mice were terminated for further postmortem evaluation four hours post injection. Xenografts, organs and fluids were harvested and macroscopically imaged using the PEARL scanner (LI-COR Biosciences) to determine ex vivo tumor uptake and biodistribution. Tissues were sectioned in sequential slides, and tracer fluorescence was imaged using an Odyssey CLx (LI-COR Biosciences). Consecutive slides were processed for fluorescence microscopy, anti-SSTR_2_ (AC-162RUO), and H&E staining as described above.

Statistical significance was tested using a one-way ANOVA with Bonferroni post-hoc analysis. Fluorescence specificity values are reported as mean ± SEM. The TBR was defined as the ratio of the mean fluorescence intensity (MFI) of the tumor, and the MFI of brain tissue. TBRs are shown as mean with 95% confidence intervals in brackets.

### Post-mortem intra-operative molecular fluorescence guided surgery

One H69 xenograft bearing mouse was intravenously injected with 800CW-TATE for post-mortem fluorescence guided surgery. The xenograft was first resected using white light guidance, followed by fluorescence guided resection of residual tissue with the SurgVision (SurgVision BV, ‘t Harde). The TBR was calculated by dividing the MFI of the tumor by the MFI of surrounding muscle tissue.

### Analyses of human meningioma specimens

Ten meningioma specimens resected between 2006 and 2012 were available, and most of these have been described previously: two WHO grade III meningiomas have been added to the current dataset [7]. Frozen sections were incubated with 10^–6^ M 800CW-TATE for one hour, mounted with Prolong Antifade, and imaged using an inverted microscope. Sequential sections were stained with either H&E or anti-SSTR_2_ (MAB4224). For whole specimen analysis, two freshly resected meningioma specimen were cut into 3 mm sections and incubated for four hours using DMEM/F12, supplemented with 1% penicillin/streptomycin and 5 × 10^–8^ M 800CW-TATE. Sections were cut for a DAPI, H&E and anti-SSTR_2_ (AC-0162RUO) stain as described above.

## Results

### Binding affinity of 800CW-TATE

To determine binding affinity of 800CW-TATE, different tracer concentrations were applied in displacement assays with radiolabeled [^177^Lu]Lu-DOTA-TATE on H69 xenograft frozen sections. DOTA-TATE has a high binding affinity for SSTR_2_ as was shown in previous work [22]. 800CW-TATE had an IC_50_ of 72 nM and unlabeled DOTA-TATE, as a control, had an IC_50_ of 2.5 nM. The IC_50_ of 800CW-TATE was considered sufficient to continue tracer evaluation. (Online Resource 2).

### Tumor uptake and biodistribution of 800CW-TATE in mice

**Fig. 1:**
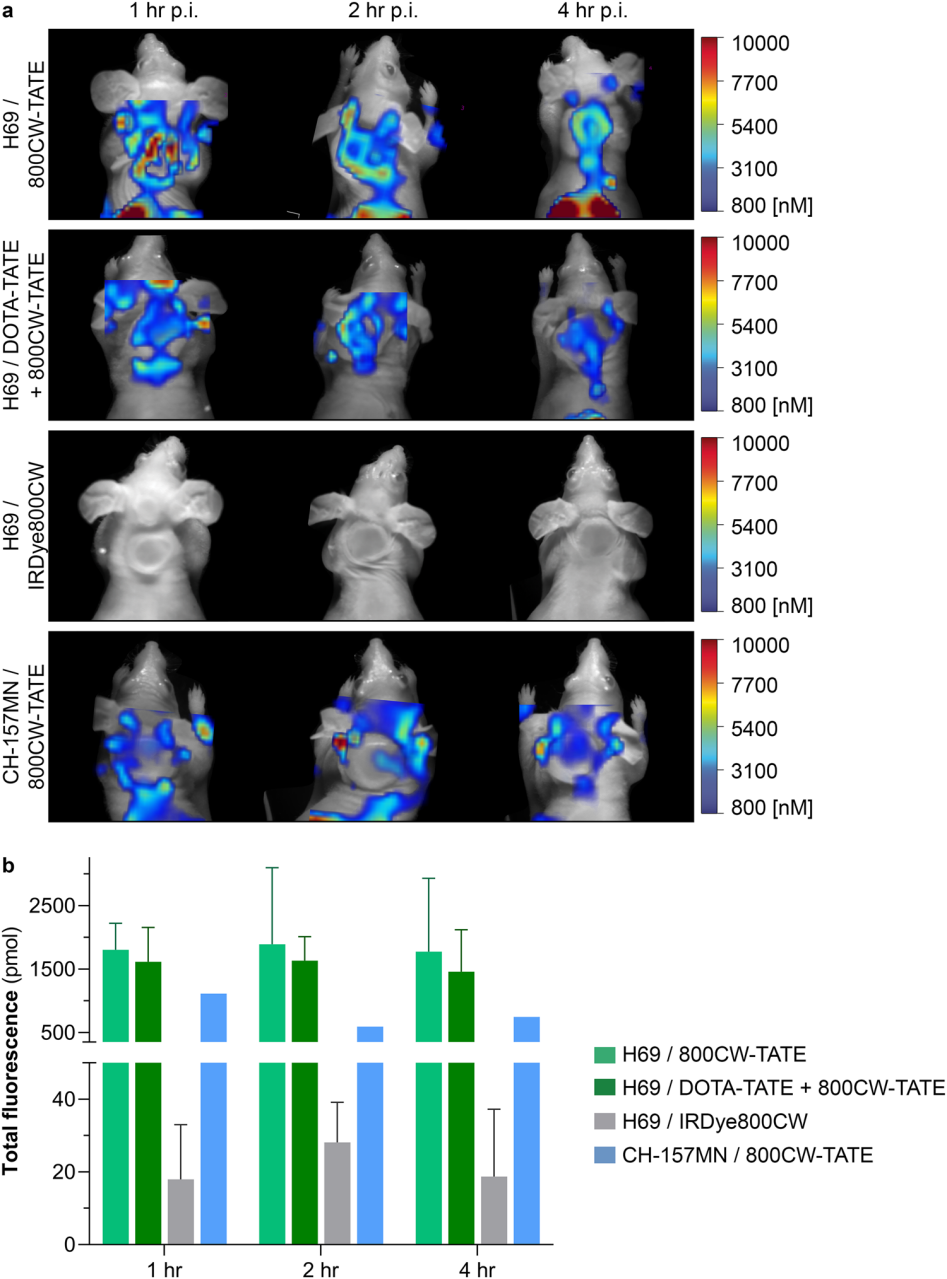
In vivo optical imaging of 800CW-TATE in H69 and CH-157MN xenografted mice. **a** Representative images of H69 xenograft bearing mice injected with 800CW-TATE showed accumulation of fluorescence in the tumor after one and two hours, and stabilized four hours post injection (top panel). Imaging of SSTR_2_-positive H69 xenograft bearing mice injected with DOTA-TATE and 800CW-TATE (second panel) or IRDye800CW (third panel), and SSTR_2_-negative CH-157MN xenograft bearing mice injected with 800CW-TATE (bottom panel) showed reduced accumulation of fluorescence compared with mice injected with 800CW-TATE. **b** The co-administration of DOTA-TATE as a block slightly reduced 800CW-TATE fluorescence at all timepoints, possibly due to uptake of 800CW-TATE in the skin, thereby distorting the signal. CH-157MN xenografts did not show tracer uptake due to the absence of SSTR_2_ expression. IRDye800CW showed a significantly reduced tumor fluorescence in H69 xenografts

To determine in vivo tumor uptake and tissue distribution of 800CW-TATE we used H69 and CH-157MN xenograft mouse models. Tracer fluorescence was clearly detected in the H69 tumor and kidneys within one hour after injection of 800CW-TATE. After four hours, a more homogeneous uptake was observed, evidenced by lower levels of fluorescence in intratumoral hotspots (Fig. 1a, top panel).

Quantification of the fluorescent tracer signal showed stabilized tumor uptake over time, since the signal did not further increase over time (Fig. 1b). SSTR_2_ selective tumor uptake was confirmed by saturating (blocking) SSTR_2_ using DOTA-TATE, followed by 800CW-TATE administration. The tracer signal appeared reduced at all-time points in vivo, although to a small extent (Fig. 1a, second panel), which is likely caused by non-specific uptake in the mouse skin, as also described further on. Additionally, IRDye800CW (without the SSTR_2_-targeting TATE) did not show tumor-specific fluorescence. (Fig. 1a, third panel).

Surprisingly, 800CW-TATE showed much less tumor fluorescence in CH-157MN xenografts compared with H69 xenografts. (Fig. 1a, bottom panel) This is explained by the absence of SSTR_2_ expression in CH-157MN xenografts, which was unexpected since in vitro cultured cells did have SSTR_2_ expression. (Online Resource 3) The CH-157MN model thus provided an additional negative control for SSTR_2_-mediated tumor uptake. Moreover, it also demonstrated that the 800CW-TATE signal likely originated from the skin covering the tumor, thereby distorting the in vivo tumor signal.

**Fig. 2:**
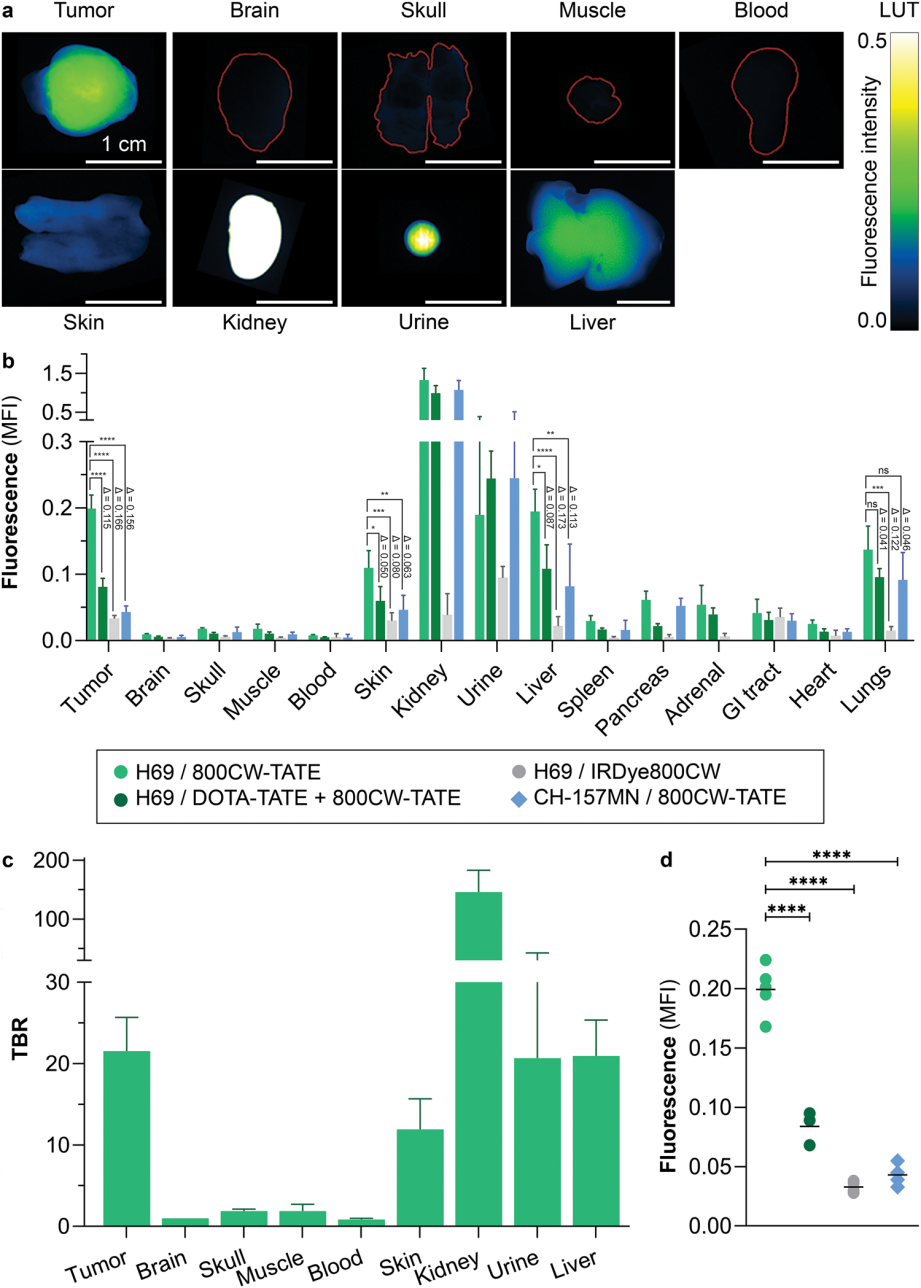
Ex vivo optical imaging of resected tissues from H69 or CH-157MN xenograft bearing mice. **a** H69 xenografted mice treated with 800CW-TATE were examined for tracer tissue distribution four hours post-injection. Fluorescence uptake was measured with the PEARL and demonstrated uptake mainly in the tumor, skin, kidneys, urine, and liver. In contrast, the fluorescence intensity was (very) low in the brain, skull, muscle, and blood. **b** The indicated xenografted mice were injected with 800CW-TATE in absence or presence of DOTA-TATE or with non-targeted IRDye800CW. Quantification of fluorescence intensity revealed the indicated biodistribution. **c** Tissue-to-brain ratios for 800CW-TATE were calculated for the indicated tissues, with a TBR of 21.1. **d** Tumor fluorescence significantly decreased in the groups co-administrated with DOTA-TATE, the groups administered with IRDye800CW carboxylate, and in CH-157MN xenografts (p < 0.0001). *ns*, not significant; Δ, difference

The biodistribution of 800CW-TATE was determined ex vivo in resected xenograft and mice tissues. Fluorescent imaging showed that 800CW-TATE mainly accumulated in H69 xenografts, kidneys, urine, liver, and to a lesser extent in the skin (Fig. 2a, b). Fluorescence was very low or absent in the brain, skull, muscle and blood (Fig. 2a, b; Online Resource 4). This was also reflected by calculating the TBR, which was 21.1 (16.4–26.7). The TBR of other relevant structures considering the anatomical localization of meningiomas was high as well. (Fig. 2c).

Tracer uptake specificity could be determined by comparing tumor fluorescence intensity between treatment groups. The DOTA-TATE block resulted in a 57.8  ±  5.2% reduction in tracer uptake. Furthermore, compared with 800CW-TATE-injected H69 xenograft bearing mice, IRDye800CW carboxylate-injected H69 xenografted mice had 83.4  ±  4.8% reduction, and SSTR_2_ negative CH-157MN xenograft bearing mice showed a reduction of 78.4  ±  4.8% tumor uptake. (Fig. 2d).

**Fig. 3:**
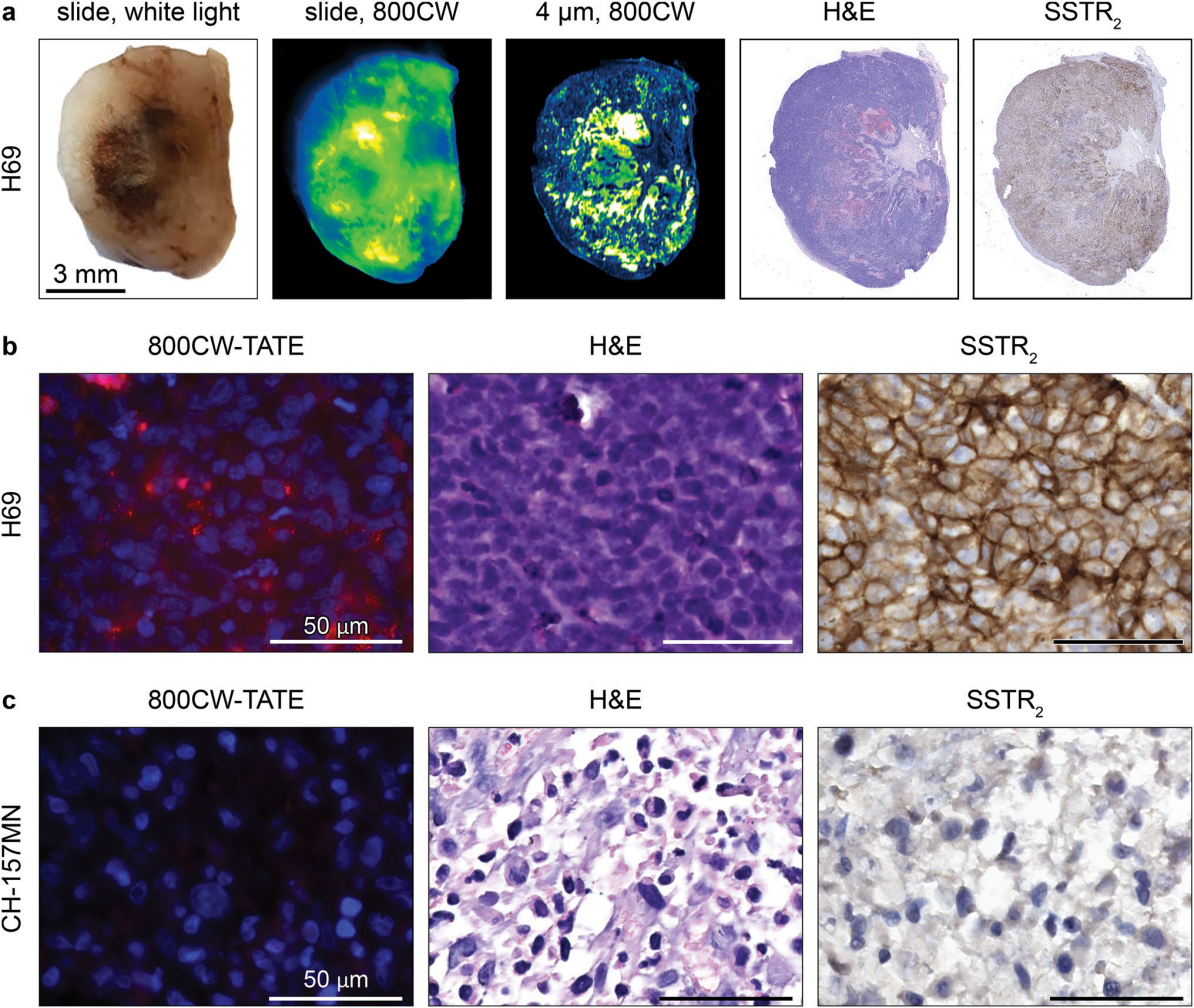
800CW-TATE uptake and SSTR_2_ expression in xenografts. **a** Macroscopic overview of a H69 tumor slice as white light image, 800CW Odyssey CLx scan of the same slice and 800CW Odyssey CLx scan of a 4 µm slide, H&E, and SSTR_2_ counterstain. **b** 800CW-TATE fluorescence and SSTR_2_ expression are homogenous throughout the tumor. Micrographs of a H69 tumor slice incubated with 800CW-TATE and stained with H&E and anti-SSTR_2_ revealed a membranous and cytoplasmatic pattern for both 800CW-TATE and SSTR_2_. **c** CH-157MN xenograft showed low 800CW-TATE fluorescence and absent SSTR_2_ expression

At the macroscopic level a homogenous tracer distribution in H69 xenografts was demonstrated after tumor resection and subsequent imaging (Fig. 3a). In the same xenograft, fluorescence microscopic analyses showed presence of 800CW-TATE mostly at the cell membrane and cytoplasm. In addition, membranous SSTR_2_ expression was confirmed by immunohistochemistry (IHC; Fig. 3b). Although CH-157MN cells showed SSTR_2_ expression in vitro (Online Resource 3), CH-157MN xenografts showed no SSTR_2_ expression, which correlated with the low fluorescence intensity observed in these xenografts (Fig. 3c).

### Exploring MFGS on post-mortem mice

**Fig. 4:**
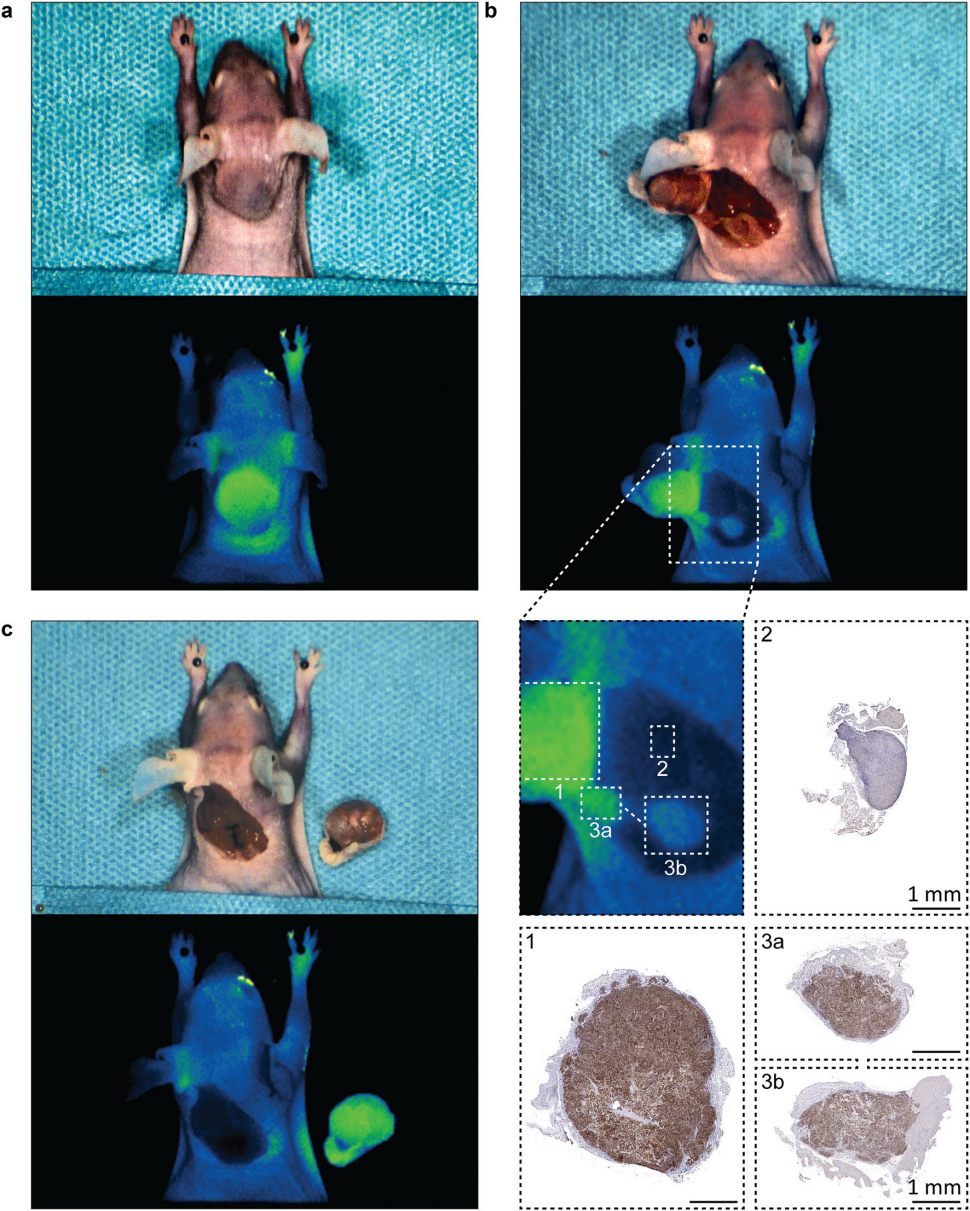
Post-mortem MFGS on H69 xenografted mouse. Mouse bearing a H69 xenograft injected with 800CW-TATE underwent post-mortem MFGS. The tumor was visualized prior (**a**) and during (**b**) xenograft removal. Small pieces of xenograft were initially undetected using white light guidance but could be readily observed using fluorescence. Fluorescent tissue showed SSTR_2_ expression, whereas non-fluorescent background tissue did not. Post resection, the wound bed shows low fluorescence intensity (**c**), with a TBR of 3.2

To explore the use of 800CW-TATE in MFGS, we mimicked the procedure on a H69-xenografted mouse post-mortem. Tumor tissue could be distinguished during all steps of xenograft resection using fluorescence guidance. (Fig. 4) The TBR was 3.2. Using white light guidance, the tumor was almost completely resected. The remaining xenograft tissue could be observed using fluorescence guidance. Histological examination showed the resected tumor expressed SSTR_2_, whereas non-fluorescent non-tumorous background muscle tissue showed no SSTR_2_ expression, in line with absence of fluorescence. (Fig. 4b).

### 800CW-TATE binding to human meningioma samples

**Fig. 5:**
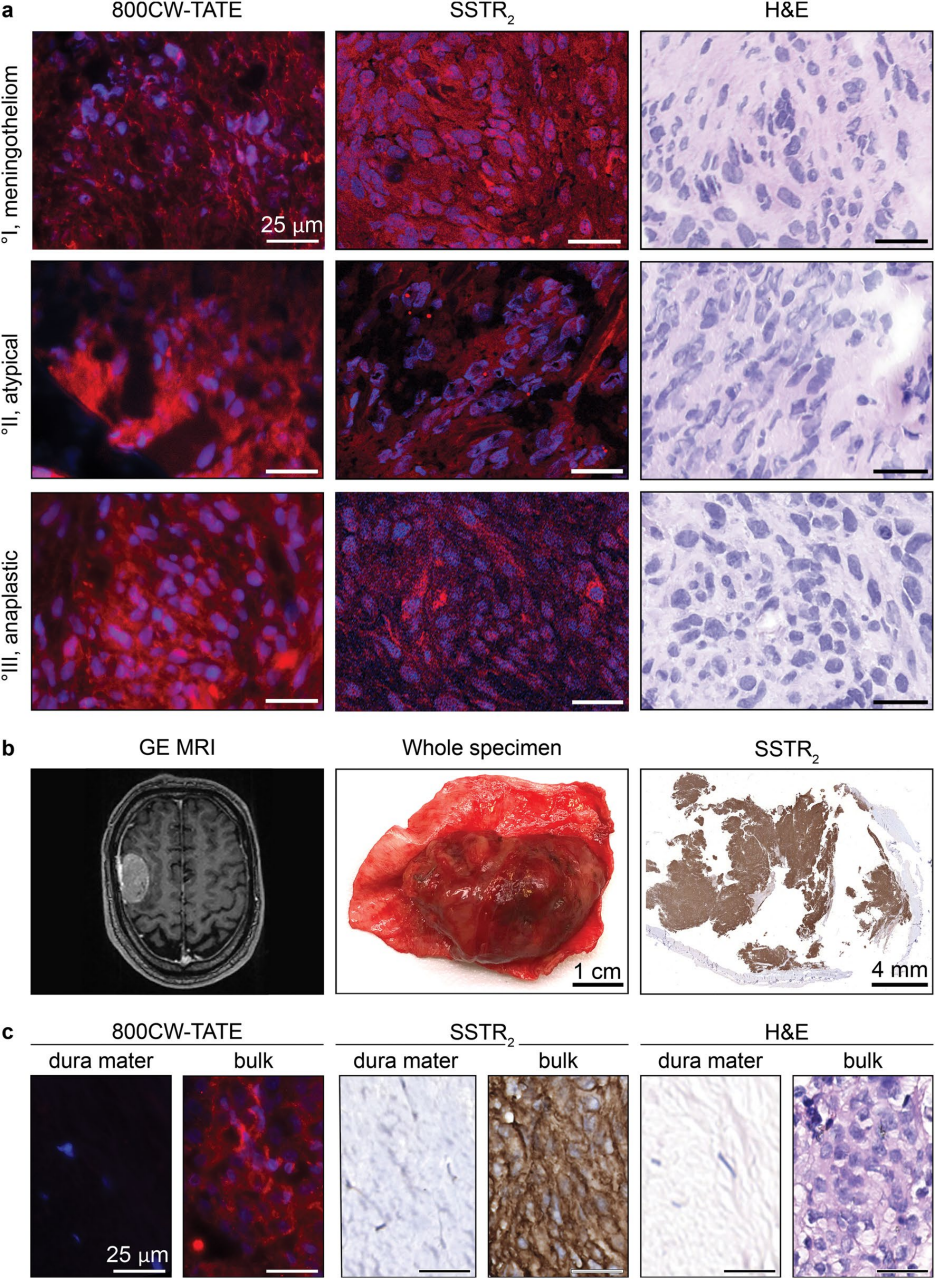
800CW-TATE uptake in clinical meningioma specimens. **a** Ten frozen meningioma specimens representing the different WHO grades were incubated with 800CW-TATE. Micrographs of sequential sections stained for 800CW-TATE (left panel), SSTR_2_ (middle panel), and H&E (right panel). WHO I (top row), WHO II (middle row), and WHO III (bottom row) are depicted for each staining. All meningioma sections stained positive for SSTR_2_ and showed 800CW-TATE fluorescence regardless of WHO grade, with a membranous and cytoplasmatic staining pattern. A convexity meningioma revealed by GE MRI in a 71-year-old male (**b**, left panel) was resected (**b**, middle panel). After incubation with 800CW-TATE, the tumor bulk fluoresced, whereas the dura did not (**c**). SSTR_2_ expression determined by IHC showed a strong membranous expression in the tumor bulk, but was absent in the dura mater, in concordance with tracer fluorescence (**b**, right panel; **c**)

The binding properties of 800CW-TATE were determined on ten frozen meningioma patient samples. The meningioma specimens, consisting of four WHO grade I, three WHO grade II and three WHO grade III samples, were evaluated for SSTR_2_ expression. Online Resource 5 shows patient characteristics and SSTR_2_ scores based on previous immunohistochemical data [7]. Incubation with 800CW-TATE showed fluorescence in all meningioma samples with membranous and cytoplasmic staining patterns. (Fig. 5a, left column) Consecutive sections were stained for SSTR_2_, revealing overall SSTR_2_-positive staining at the same cellular localization as the 800CW-TATE counterstain. (Fig. 5a, middle column) Fluorescence intensity of 800CW-TATE was variable and did not correlate with WHO grades, but showed a positive trend between SSTR_2_ expression levels and fluorescence intensity.

Moreover, fresh meningioma samples (Online Resource 5) containing adjacent non-tumorous dura mater as well as meningioma tissue were analyzed for tracer binding and SSTR_2_ expression in order to evaluate selectivity for meningioma tissue. Preoperative imaging showed a convexity meningioma (Fig. 5b, left panel), which is depicted in the middle panel of Fig. 5b as a fresh specimen post resection. SSTR_2_ expression was strong in the meningioma bulk, but absent in adjacent dura mater which was non-tumorous confirmed by histopathology. (Fig. 5b, right panel; Online Resource 5) The same pattern was observed for 800CW-TATE. (Fig. 5c) These results were comparable in the tested fresh samples.

## Discussion

In this study we have generated a SSTR_2_-specific tracer, 800CW-TATE, and evaluated its potential for application in MFGS of meningiomas in xenograft mice models and clinical meningioma samples. The binding affinity of 800CW-TATE was sufficient to proceed to tracer characterization. Displacement assays showed that DOTA-TATE has a higher affinity for SSTR_2_ than 800CW-TATE, however IC_50_ values for both were in the nanomolar range. The lower affinity for 800CW-TATE may be due to steric hindrance, an altered configuration and/or electrical charge of the tracer [23–25].

In this proof-of-principle study, xenografts of lung cancer H69 cells served as a SSTR_2_-positive tumor model [17–19]. The xenografts were heterotopically inoculated, creating a model that remained representative for meningiomas, because these intracranial tumors are located outside the blood–brain-barrier (BBB) and are all SSTR_2_ positive. Surprisingly, CH-157MN xenografts did not show SSTR_2_-expression, while CH-157MN cell cultures did express SSTR_2_ in line with our previous experience [7]. Thus, the H69 xenograft model provided a suitable model for SSTR_2_ targeting, whereas the CH-157MN xenograft model eventually served as a SSTR_2_-negative control in this study. Clearly, the use of the CH-157MN xenograft model would have been ideal to further illustrate the suitability of 800CW-TATE for visualizing meningioma tissue, however, as an alternative strategy fresh frozen meningioma specimen were used, as discussed in more detail below.

In vivo, binding of 800CW-TATE appeared to be SSTR_2_-mediated, as fluorescence uptake determined ex vivo on resected xenograft significantly decreased after a DOTA-TATE block and fluorescence was clearly lower in the CH-157MN xenografts. Although the block was not complete, this could be due to tracer binding to necrotic tissue, which we are currently investigating in a separate project. Comparison of tumor specific binding of 800CW-TATE to other IRDye800CW-coupled tracers is complicated due to the use of targeting vectors, imaging modalities and animal models described in literature. Previously, bevacizumab-IRDye800CW showed a 65% higher fluorescence signal compared with a non-targeted IgG-IRDye800CW control [26], which was in line with other studies applying various antibody-IRDye800CW labelled tracers [27–29]. Furthermore, several in vivo studies using octreotate-based tracers showed that tumor uptake of both [^177^Lu]Lu-DOTA-TATE and [^213^Bi]Bi-DOTA-TATE was blocked after the administration of DOTA-TATE [17, 18]. Renal clearance of the tracer was observed by high fluorescence levels in kidneys and urine. In addition, tracer uptake was determined in liver, lungs, brain, muscle and blood. Uptake in these organs was mostly comparable to previously published reports applying [^111^In]In-DOTA-TATE, [^213^Bi]Bi-DOTA-TATE, [^111^In]In-DTPA-TATE or Cy5-[^111^In]In-DTPA-TATE [17–19]. Lastly, IRDye800CW-carboxylate-treated H69 xenograft bearing mice had very low uptake of the fluorescent dye, indicating that tumor fluorescence is not related to freely circulating dye in our study. This is in agreement with other reports showing no tumor-specific uptake of IRDye800CW in a colon cancer model and orthotopic glioma model [30, 31].

The ex vivo TBR of 21.1 in our present experiments is encouraging for future clinical translation, based on our experience in both preclinical and clinical intra-operative imaging settings [32, 33]. Fluorescence was low in neurosurgical background tissues such as brain, skull and muscle, allowing for clear distinction and visualization of tumor tissue. This is highly relevant when using 800CW-TATE for resection of intracranial meningioma. Tracer fluorescence detected in the skin is usually not of importance during neurosurgery because the skin flap is retracted out of the operative area and draped. However, the relevance of background fluorescence from the skin in patients with meningioma-invasion into the skull and (sub) cutaneous tissues should be evaluated in future clinical MFGS studies. Our post-mortem MFGS experiment on a H69 xenograft bearing mouse achieved a TBR of 3.2 and small xenograft residues could be easily detected. In our experience, various clinical imaging systems for fluorescence guided surgery require a TBR > 2.0 to be able to be of value in a clinical setting.

Lastly, the clinical meningioma specimens (representing different WHO grades) all expressed SSTR_2_ and showed 800CW-TATE-specific fluorescence staining. Importantly, 800CW-TATE could distinguish meningioma from dura mater on freshly resected specimens. The observed variation in fluorescence intensity of both stainings varied among WHO grades, which is not in line with larger sample sizes available in literature and our previous results where we found no significant differences in SSTR_2_-expression among WHO grades [7–11]. Nevertheless, the current results illustrate that 800CW-TATE binds to all meningioma grades. Future in-human trials will further elucidate the relationship between SSTR_2_ expression and 800CW-TATE fluorescence.

SSTR_2_-targeted tracers have already been applied for diagnostics and treatment of meningiomas. For example, in tumor imaging ^111^In-octreotide SPECT in combination with MRI has been successfully used to differentiate meningioma from other intracranial lesions [34]. ^68^ Ga-DOTA-TOC- and ^68^ Ga-DOTA-TATE-PET/MRI provided better imaging quality during follow-up of intracranial meningiomas and improved the ability of detection of residual meningioma or tumor recurrence after initial treatment [35, 36]. Ongoing trials further investigate diagnostic and follow-up imaging feasibility of SSTR_2_-targeted radiopeptides in meningioma (NCT04298541, NCT03953131). Furthermore, peptide receptor radiotherapy with ^90^Y- and ^177^Lu-DOTA-TOC has resulted in patients with inoperable meningioma reaching stable disease [37]. Currently, ongoing phase II trials with among others ^177^Lu-DOTA-TATE (Lutathera, NCT04082520), ^90^Y-DOTA-TOC and ^90^Y-DOTA-TATE (NCT03273712) will further demonstrate the value of this treatment modality for inoperable, progressive meningiomas. Our next step in the development of 800CW-TATE for MFGS in meningiomas will be the clinical evaluation of this tracer in patients with intracranial meningiomas, aiming to add another SSTR_2_-directed approach to the treatment of this type of tumors.

Dural or bone thickening may be indicative of meningioma invasion, but can also be caused by vascular congestion or reactive tissue changes. Furthermore, meningioma invasion may be present in these adjacent tissues without causing macroscopically detectible differences using white light. This can lead to a (yet undetected) subtotal resection, which is a major risk factor for recurrence [2]. Currently applied dyes, such as 5-ALA, ICG and fluorescein, have a too low sensitivity and/or specificity to distinguish meningioma invasion from reactive changes or even from normal tissue of the adjacent dura and bone. 800CW-TATE targets SSTR_2_ which is not expressed in normal bone or dura mater. Thus, in a future clinical study we expect that with the aid of this tracer and technique, not only small tumor remnants or satellite lesions will be detected during primary or recurrent tumor resection, but meningioma invasion status of adjacent soft and bone tissue will be determined more accurately as well.

## Conclusions

800CW-TATE appears to be a promising fluorescent dye for MFGS of intracranial meningiomas. It has the potency to aid in the safer and completer resection, which is especially relevant in the high grade meningiomas or in meningiomas located in difficult anatomical locations.

### Future perspectives

With these encouraging preclinical results, a dose-finding clinical trial is feasible. Indeed, both octreotate and IRDye800CW have been applied numerous times in humans, increasing the chances of successful clinical translation [16, 38, 39]. For a first in-human trial, a microdosing study with 800CW-TATE in a small number of patients would be the next step. In line with our preclinical study results regarding timing of tracer administration, tumor uptake and imaging, intravenous tracer administration in patients could take place approximately four hours prior to surgical incision, but can be adjusted to obtain optimal TBRs. Altogether, 800CW-TATE shows a favorable biodistribution for application in a neurosurgical setting, with the ultimate goal of improving curative resection rates in patients with intracranial meningioma.

## Supplementary Information

Below is the link to the electronic supplementary material.

Supplementary file1 Chemical characteristics of 800CW-TATE. In order to form the investigated tracer 800CW-TATE, Tyr3-octreotate (depicted in black) was linked with Ac-lysine (depicted in blue) to the fluorescent dye IRDye800CW (depicted in green) (TIF 828 kb).

Supplementary file2 Concentration-binding curves of [177Lu]Lu-DOTA-TATE to SSTR2 when blocked with DOTA-TATE or 800CW-TATE. In vitro [177Lu]Lu-DOTA-TATE blocking assay on H69 xenograft slides using a concentration range of either DOTA-TATE and 800CW-TATE, showing a receptor affinity for SSTR2 in the nanomolar range: 2.5 nM and 72 nM, respectively (TIF 408 kb).

Supplementary file3 SSTR2 expression in CH-157MN and H69 cell lines in vitro. Both cell lines were SSTR2 positive in vitro prior to tumor inoculation (overview/top panel). CH-157MN mainly showed SSTR2 expression at the cell membrane, as evidenced by the honeycomb staining pattern. SSTR2 expression in the H69 cell line was more pronounced in the cytoplasm. (zoom/bottom panel) (TIF 13368 kb).

Supplementary file4 800CW-TATE and SSTR2 micrographs of tissues of interest. Tissues were harvested from mice injected with 800CW-TATE. The brain, skull, muscle and liver showed (almost) no 800CW-TATE fluorescence and low SSTR2 expression. 800CW-TATE signal in the skin is mainly located in the intermediate layer. In the kidneys, fluorescence was high. Scale bar represents 25 µm (TIF 11613 kb).

Supplementary file5 (DOCX 18 kb)

Supplementary file6 (PDF 92 kb)

## Data Availability

The datasets generated during and/or analyzed during the current study are available from the corresponding author on reasonable request.

## Abbreviations

800CW-TATE: Ac-Lys^0^ (IRDye800CW)Tyr^3^-octreotate
BBB: Blood–brain-barrier
DAB: 3,3-Diaminobenzidine
DAPI: 4′,6-Diamidino-2-phenylindole
DOTA: 1,4,7,10-Tetraazacyclododecane-1,4,7,10-tetraacetic acid
DOTA-TATE: DOTA-Tyr^3^-octreotate
DOTA-TOC: DOTA-Phe^1^-Tyr^3^-octreotide
FCS: Fetal calf serum
FFPE: Formalin fixed paraffin embedded
GE MRI: Gadolinium enhanced magnetic resonance imaging
H&E: Hematoxylin & eosin
HRP: Horse radish peroxidase
IHC: Immunohistochemistry
MFGS: Molecular fluorescence guided surgery
MFI: Mean median fluorescence intensity
PBS: Phosphate buffered saline
SEM: Standard error of mean
SSTR_2_: Somatostatin receptor subtype 2
TBR: Tumor-to-background ratio
WHO: World health organization
