# Correction to: Evaluation of Ac-Lys^0^(IRDye800CW)Tyr^3^-octreotate as a novel tracer for SSTR_2_-targeted molecular fluorescence guided surgery in meningioma

**DOI:** 10.1007/s11060-021-03769-9

**Published:** 2021-05-20

**Authors:** Bianca M. Dijkstra, Marion de Jong, Marcus C. M. Stroet, Fritz Andreae, Sebastiaan E. Dulfer, Marieke Everts, Schelto Kruijff, Julie Nonnekens, Wilfred F. A. den Dunnen, Frank A. E. Kruyt, Rob J. M. Groen

**Affiliations:** 1 Department of Neurosurgery, University of Groningen, University Medical Center Groningen, Hanzeplein 1, P.O. Box 30.001, 9700 VB Groningen, The Netherlands; 2 Department of Radiology and Nuclear Medicine, Erasmus MC, Rotterdam, The Netherlands; 3 Department of Molecular Genetics, Oncode Institute, Erasmus MC, Rotterdam, The Netherlands; 4 piCHEM Forschungs und EntwicklungsGmbH, Raaba-Grambach, Graz, Austria; 5 Department of Medical Oncology, University of Groningen, University Medical Center Groningen, Groningen, The Netherlands; 6 Department of Surgery, University of Groningen, University Medical Center Groningen, Groningen, The Netherlands; 7 Department of Pathology and Medical Biology, University of Groningen, University Medical Center Groningen, Groningen, The Netherlands

## Correction to: Journal of Neuro-Oncology 10.1007/s11060-021-03739-1

The **Funding** section in the initial online article was incomplete. The original article has been corrected.

