# Supplementary figures and images for: Evaluation of Ac-Lys0(IRDye800CW)Tyr3-octreotate as a novel tracer for SSTR2-targeted molecular fluorescence guided surgery in meningioma
Source: J Neurooncol. 2021 Mar 26;153(2):211–22. doi: 10.1007/s11060-021-03739-1 (PMC8211583; doi:10.1007/s11060-021-03739-1)

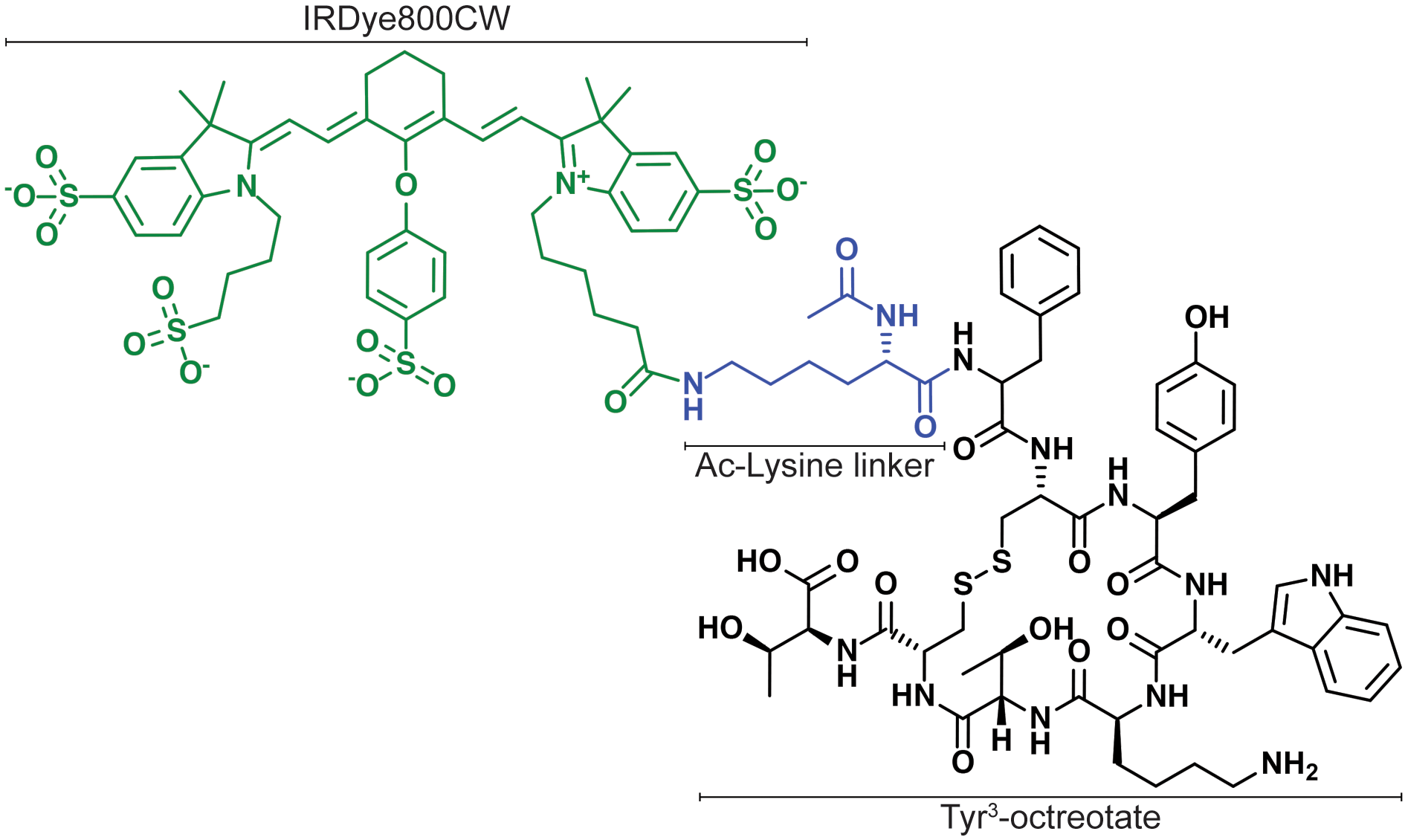

Supplement: Supplementary file 1 — Supplementary file1 Chemical characteristics of 800CW-TATE. In order to form the investigated tracer 800CW-TATE, Tyr3-octreotate (depicted in black) was linked with Ac-lysine (depicted in blue) to the fluorescent dye IRDye800CW (depicted in green) (TIF 828 kb). [file 11060_2021_3739_MOESM1_ESM.tif]

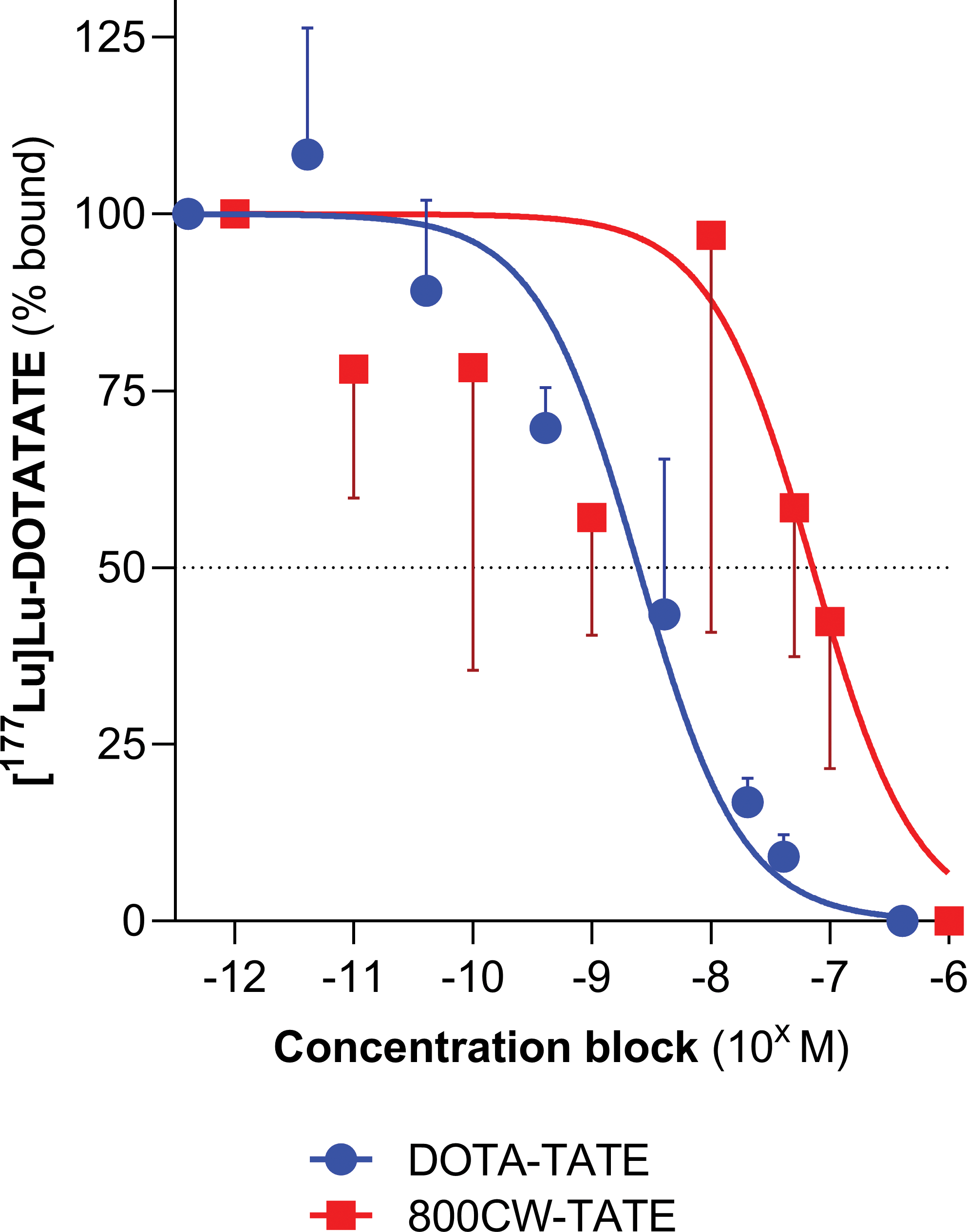

Supplement: Supplementary file 2 — Supplementary file2 Concentration-binding curves of [177Lu]Lu-DOTA-TATE to SSTR2 when blocked with DOTA-TATE or 800CW-TATE. In vitro [177Lu]Lu-DOTA-TATE blocking assay on H69 xenograft slides using a concentration range of either DOTA-TATE and 800CW-TATE, showing a receptor affinity for SSTR2 in the nanomolar range: 2.5 nM and 72 nM, respectively (TIF 408 kb). [file 11060_2021_3739_MOESM2_ESM.tif]

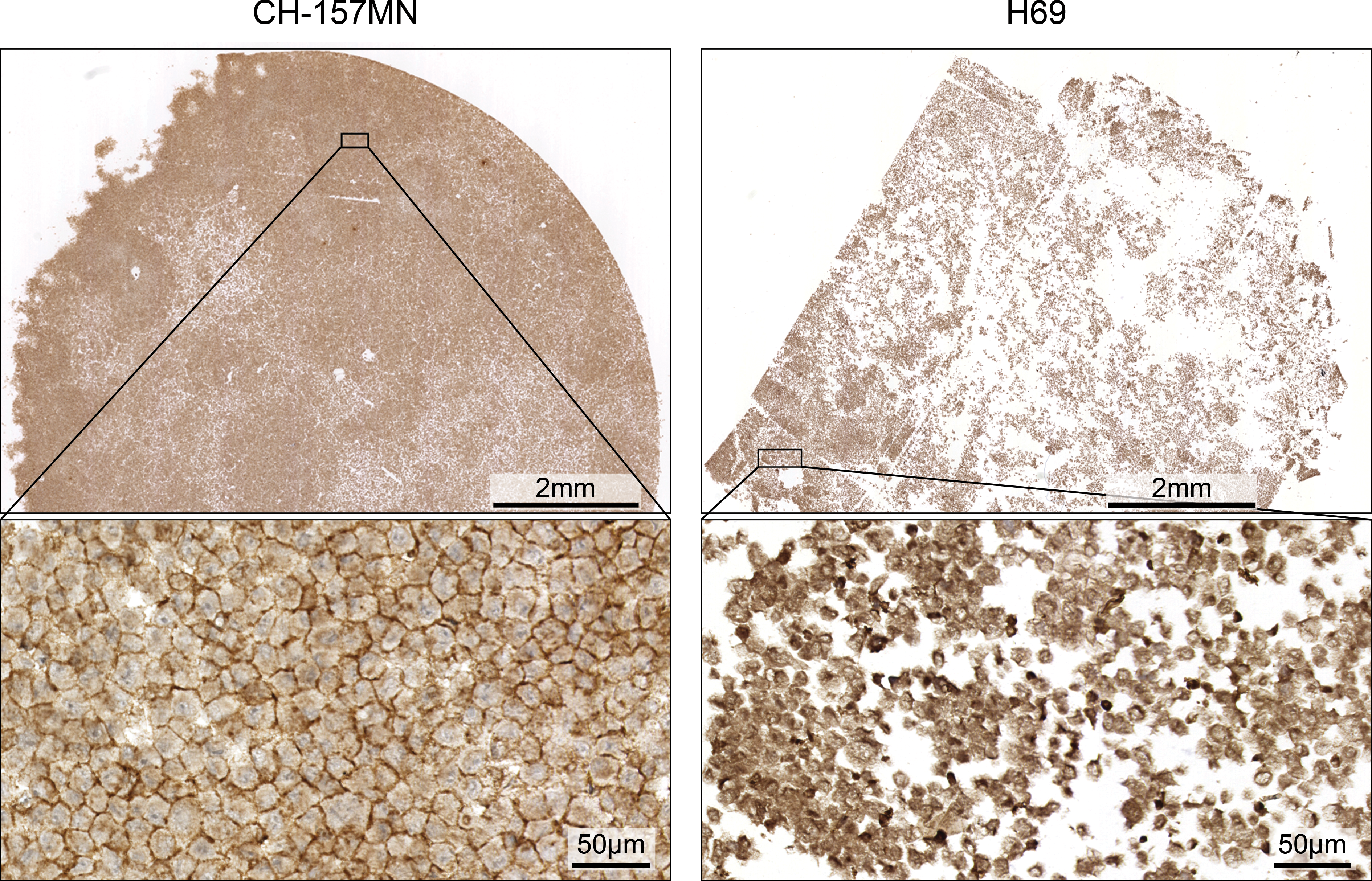

Supplement: Supplementary file 3 — Supplementary file3 SSTR2 expression in CH-157MN and H69 cell lines in vitro. Both cell lines were SSTR2 positive in vitro prior to tumor inoculation (overview/top panel). CH-157MN mainly showed SSTR2 expression at the cell membrane, as evidenced by the honeycomb staining pattern. SSTR2 expression in the H69 cell line was more pronounced in the cytoplasm. (zoom/bottom panel) (TIF 13368 kb). [file 11060_2021_3739_MOESM3_ESM.tif]

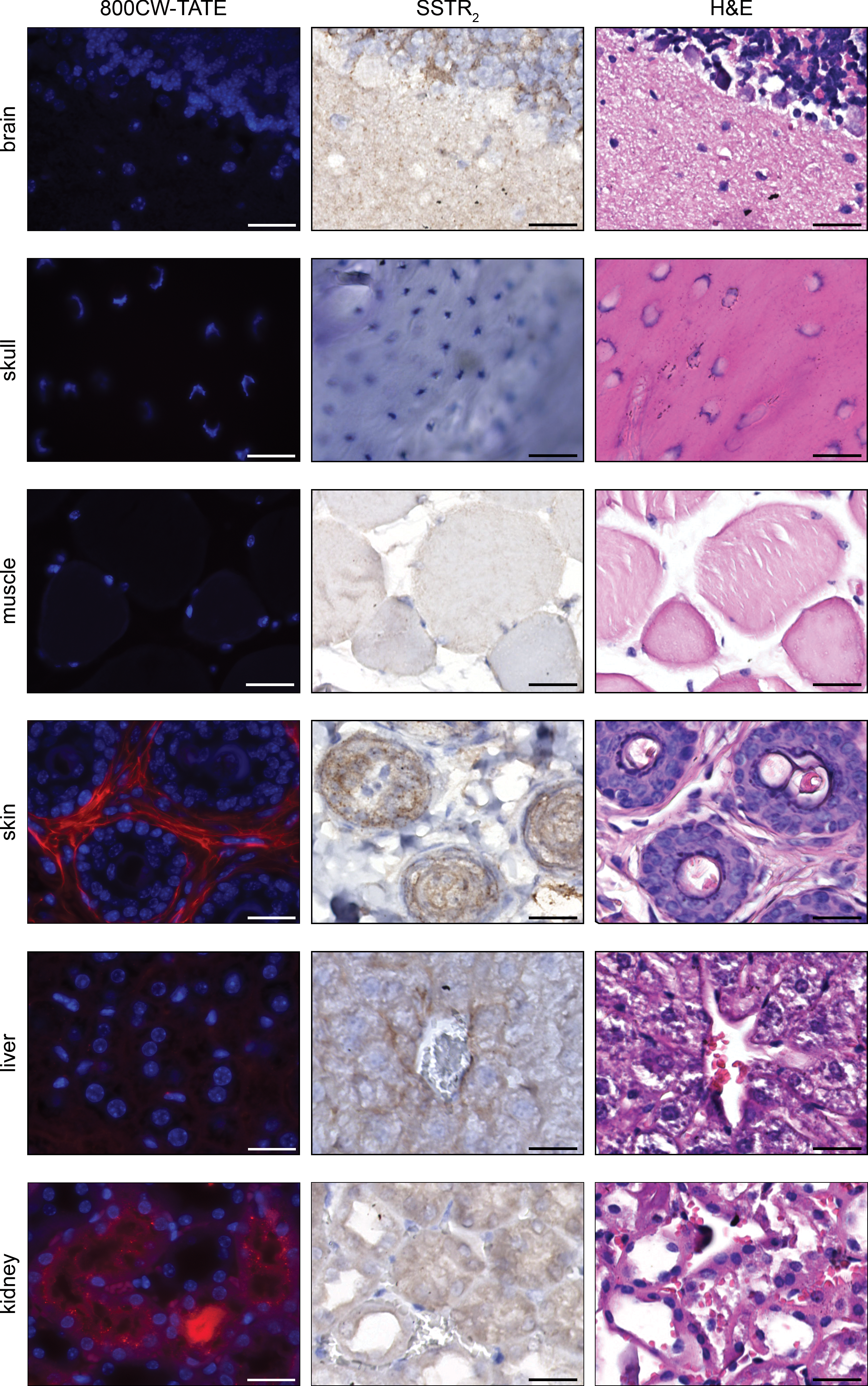

Supplement: Supplementary file 4 — Supplementary file4 800CW-TATE and SSTR2 micrographs of tissues of interest. Tissues were harvested from mice injected with 800CW-TATE. The brain, skull, muscle and liver showed (almost) no 800CW-TATE fluorescence and low SSTR2 expression. 800CW-TATE signal in the skin is mainly located in the intermediate layer. In the kidneys, fluorescence was high. Scale bar represents 25 µm (TIF 11613 kb). [file 11060_2021_3739_MOESM4_ESM.tif]
