# Supplementary material for: Evaluation of Ac-Lys0(IRDye800CW)Tyr3-octreotate as a novel tracer for SSTR2-targeted molecular fluorescence guided surgery in meningioma
Source: J Neurooncol. 2021 Mar 26;153(2):211–22. doi: 10.1007/s11060-021-03739-1 (PMC8211583; doi:10.1007/s11060-021-03739-1)
Supplement: Supplementary file 5 — Supplementary file5 (DOCX 18 kb) [file 11060_2021_3739_MOESM5_ESM.docx]

| Sex, age (yr) | WHO grade and subtype | SSTR_2_ score^a^ bulk | SSTR_2_ score^a^ dura |
| --- | --- | --- | --- |
| F, 77 | I / Meningotheliomatous | 3 | ND |
| F, 38 | I / Meningotheliomatous | 1 | ND |
| F, 75 | I / Transitional | 2 | ND |
| F, 64 | I / Fibromatous | 3 | ND |
| F, 61 | II / Atypical | 3 | ND |
| M, 63 | II / Atypical | 2 | ND |
| F, 82 | II / Atypical | 3 | ND |
| M, 43 | III / Anaplastic | 3 | ND |
| F, 72 | III / Anaplastic | 1 | ND |
| F, 64 | III / Anaplastic | 1 | ND |
| F, 58 | I / Transitional | 3 | 0 |
| M, 71 | II / Atypical | 3 | 0 |

**Online Resource 5** Table with baseline characteristics of the tested meningioma frozen sections.

ND, not determined; ^a^ SSTR_2_ score ranged from 1 to 3, applying the following staining scoring system [7]: negative, (0); weak/focal staining, (1); moderate/diffuse staining, (2); strong/diffuse staining, (3)
