# Supplementary material for: Evaluation of Ac-Lys0(IRDye800CW)Tyr3-octreotate as a novel tracer for SSTR2-targeted molecular fluorescence guided surgery in meningioma
Source: J Neurooncol. 2021 Mar 26;153(2):211–22. doi: 10.1007/s11060-021-03739-1 (PMC8211583; doi:10.1007/s11060-021-03739-1)
Supplement: Supplementary file 6 — Supplementary file6 (PDF 92 kb) [file 11060_2021_3739_MOESM6_ESM.pdf]

Supplemental methods and materials

Cell culture

Small cell lung cancer NCI-H69 (ECACC) cells were maintained in RPMI-1640 medium (Thermo Fisher Scientific, Breda, The Netherlands) supplemented with 10% fetal calf serum (FCS; Bodinco BV, Alkmaar, The Netherlands). [1–3] The meningioma cell line CH-157MN was kindly provided by dr. Gillespie (University of Alabama School of Medicine, Birmingham, Ala) and maintained in DMEM/F12 (Thermo Fisher Scientific) supplemented with 7% FCS. [4] Cells were maintained at 37°C in a humidified atmosphere with 5% CO_2.

Immunohistochemistry on formalin fixed paraffin embedded (FFPE) sections

$SSTR_2$ expression in H69 and CH-157MN cells *in vitro* was determined and confirmed by immunohistochemistry (IHC), by analyzing sections of Cellient blocks stained with the primary antibody mouse-anti-$SSTR_2$ (MAB4224, R&D systems, Abingdon, United Kingdom; 1:100). Sections of 4 µm were deparaffinized in xylene and rehydrated in ethanol. Tris/EDTA buffer (pH 9.0) was used for antigen retrieval for seven minutes in a microwave. Endogenous peroxidase was blocked by incubation with 0.3% hydrogen peroxidase for 30 minutes. The primary antibody $SSTR_2$ (MAB4224, R&D systems; 1:100) was incubated for one hour at room temperature. Consecutively, slides were washed, incubated for 30 minutes with a secondary (goat anti-mouse horse-radish-peroxidase (HRP), DAKO; 1:75) and tertiary (rabbit anti-goat HRP, DAKO; 1:75) antibody. All sections were incubated with 3,3-diaminobenzidine (DAB) solution for 10 min and finally counterstained with hematoxylin for 2 min. They were dehydrated in ethanol, mounted with a cover slip, and scanned using the NanoZoomer 2.0 HT multi-slider scanner (Hamamatsu).

FFPE tissue obtained from animal studies was sliced in 4 µm sections. Separate consecutive slices were deparaffinized and used for either 4′,6-diamidino-2-phenylindole (DAPI) stain, $SSTR_2$ IHC, and hematoxylin and eosin (H&E) staining. For fluorescence microscopy, slices were mounted using ProLong® Diamond Antifade Mountant with DAPI (ThermoFisher) and imaged using an inverted microscope (DMI6000B, Leica Biosystems) with additional accessories as described previously.[32] IHC analysis of $SSTR_2$ took place as described above, with the following exceptions. A Pascal autoclave was applied for antigen retrieval. The primary antibody used was $SSTR_2$ (AC-0162RUO, SanBio, Uden, the Netherlands; 1:100), with the appropriate secondary (goat anti-rabbit HRP, DAKO; 1:75) and tertiary (rabbit anti-goat HRP, DAKO; 1:75) antibodies. [5]

H&E staining

Sections were deparaffinized in xylene and rehydrated in ethanol. Slices were incubated in hematoxylin for ten minutes, washed with tap water and incubated with eosin for two minutes. Finally, they were washed and dehydrated in ethanol, mounted with a cover slip and scanned using NanoZoomer 2.0 HT multi-slider scanner (Hamamatsu, Hamamatsu City, Japan).

Xenograft mice models

H69 cells (5 million cells) were inoculated in six to eight-week-old BALB/c-nu mice (Janvier Laboratories, Le Genest-Saint-Isle, France) in a 300 µl 1:1 mix of Matrigel (Corning, #354248) and medium. [1–3] CH-157MN cells

were inoculated by injection of 1.5 million cells in medium. In both models, the cells were subcutaneously injected between the scapulae. Tumor growth was monitored biweekly using a caliper. The Institutional Review Board of the University of Groningen approved the study and animal care complied with the Guide for the Care and Use of Laboratory Animals.

Displacement assays

Resected H69 xenografts of 500 mm^3, known to abundantly express $SSTR_2$ [1–3], were snap frozen and sliced in 10 µm frozen sections. These were washed with 0.25% BSA in washing buffer (167 mM Tris-HCl, 5 mM $MgCl_2$) for ten minutes and incubated with 10^{-9} M [^{177}Lu]Lu-DOTA-Tyr^3-octreotate ([^{177}Lu]Lu-DOTA-TATE; molar activity 53 MBq/nmol, radiometal incorporation >95% and radiochemical purity >90%; IDB Holland, Baarle-Nassau, the Netherlands) together with a concentration range of 10^{-6} to 10^{-12} M 800CW-TATE (piCHEM, Graz, Austria; Online Resource 1), or DOTA-TATE (Bachem, Torrance, CA, USA) as control. Sections were subsequently washed, and activity was detected with the Cyclone (PerkinElmer). Data was analyzed using OptiQuant 5.0 and GraphPad Prism 8 as described previously. [6]

Tracer uptake, biodistribution and imaging

Mice bearing xenografts with a volume of approximately 500 mm^3 were divided into treatment groups for imaging. The animals were anesthetized with 5% isoflurane and using 2.5% isoflurane for maintenance. Indicated doses of tracer or DOTA-TATE were retro-orbitally injected. Tumor uptake of 800CW-TATE was determined by longitudinal imaging for 1, 2, and 4 hours post tracer injection using the FMT2500 (PerkinElmer, Waltham, Massachusetts, United States). Tracers were dissolved in phosphate buffered saline (PBS). For H69 xenografted mice, five mice were intravenously injected with 800CW-TATE (3 µg, 1.36 nmol, 50 µl), three mice with DOTA-TATE (3 mg, 2.2 µmol, 100 µl) and 800CW-TATE (3 µg, 1.36 nmol, 50 µl) in three consecutive injections with five-minute intervals, and four mice were injected with IRDye800CW carboxylate (1.5 µg, 1.36 nmol, 50 µl; LI-COR Biosciences, Lincoln, Nebraska, United States). CH-157MN xenograft bearing mice were injected with 800CW-TATE (3 µg, 1.36 nmol, 50 µl). Mice were terminated by heart puncture and cervical dislocation for further postmortem evaluation. Xenografts, organs and fluids were harvested and macroscopically imaged using the PEARL scanner (LI-COR Biosciences) to determine *ex vivo* tumor uptake and biodistribution. Tumor, brain, skull, muscle, skin, kidney, and liver tissue were sectioned in sequential slides, and tracer fluorescence was imaged using an Odyssey CLx (LI-COR Biosciences). Consecutive slides were processed for fluorescence microscopy and anti-$SSTR_2$ and H&E staining as described above.

Data was analyzed using ImageJ and GraphPad Prism 8.0. Statistical significance was tested using a one-way ANOVA with Bonferroni post-hoc analysis; a P-value of ≤ 0.05 was considered statistically significant. Fluorescence specificity values are reported as mean±SEM. The TBR was defined as the ratio of the mean fluorescence intensity (MFI) of the tumor, and the MFI of brain tissue. TBRs are shown as mean with 95% confidence intervals in brackets.

Post-mortem intra-operative molecular fluorescence guided surgery

One H69 xenograft bearing mouse (500 mm^3) was intravenously injected with 800CW-TATE (3 µg, 1.36 nmol, 50 µl) for post-mortem fluorescence guided surgery. Draping was used to block fluorescence of the kidneys. The xenograft was first resected using white light guidance, followed by fluorescent guided resection of residual tissue with the SurgVision (SurgVision BV 't Harde, The Netherlands). Images were processed using ImageJ, and the TBR was calculated by dividing the MFI of the tumor by the MFI of surrounding tissue. The background MFI was measured within a spherical volume of interest positioned in an area of physiological muscle tissue.

Analyses of human meningioma specimens

Meningioma specimens resected at the University Medical Center Groningen between 2006 and 2012 were available, and most of these have been described previously: two WHO grade III meningiomas have been added to the current dataset, leading to ten analyzed samples. [5] The Institutional Review Board approved the experimental protocol and informed consent. If informed consent could not be obtained, the Code of Conduct for responsible use of human tissue was followed.

Frozen sections (4 µm) were incubated with 10^{-6} M 800CW-TATE for one hour, mounted with Prolong Antifade, and imaged using the inverted microscope as described above. Sequential sections were stained with either H&E or anti-$SSTR_2$. For the latter, the primary antibody mouse-anti-$SSTR_2$ (MAB4224, R&D systems; 1:100) incubated for one hour at room temperature, followed by incubation with the secondary antibody rabbit anti-mouse Alexa Fluor 647(A21239, Invitrogen, Carlsbad, California, United States; 1:75). Slides were mounted using ProLong Antifade and imaged using a Leica SP8 Confocal Laser Scan Microscope (Leica Biosystems, Wetzlar, Germany).

For whole specimen analysis, two freshly resected meningioma specimen were cut into 3 mm sections and transferred to a 6-well plate containing 2 ml DMEM/F12, supplemented with 1% penicillin/streptomycin and 5×10^{-8} M 800CW-TATE. The specimens were placed on an orbital shaker at 37°C and 5% CO_2. After four hours, slides were washed, fixed in formalin and embedded in paraffin. 4 µm sections were cut for a DAPI, H&E and anti-$SSTR_2$ (AC-0162RUO, 1:100) stain as described above.
